# Psychopharmacology of Methamphetamine in Relation to the United Kingdom Sentencing Guidance: Comparative Analysis with Amphetamine, Cocaine and Heroin

**DOI:** 10.3390/brainsci16060602

**Published:** 2026-05-31

**Authors:** Amira Guirguis, Arianna Giorgetti, Jegak Seo, John Martin Corkery, Fabrizio Schifano

**Affiliations:** 1 Pharmacy, Faculty of Medicine, Health and Life Science, Swansea University, Singleton Campus, The Grove, Swansea SA2 8PP, UK; 2 Department of Medical and Surgical Sciences, University of Bologna, 40121 Bologna, Italy; ari.giorgetti@gmail.com; 3 Royal College of Pharmacy, 66-68 East Smithfield, London E1W 1AW, UK; jegak.seo@rcpharm.org; 4 Psychopharmacology, Drug Misuse, and Novel Psychoactive Substances Research Unit, University of Hertfordshire, College Lane Campus, Hatfield AL10 9AB, UK; j.corkery@herts.ac.uk (J.M.C.); f.schifano@herts.ac.uk (F.S.)

**Keywords:** methamphetamine, amphetamine, cocaine, toxicity, smoked, sentencing comparator

## Abstract

**Highlights:**

**What are the main findings?**

- Methamphetamine shows distinct pharmacokinetic and pharmacodynamic properties compared with amphetamine, including greater CNS penetration and longer-lasting effects, suggesting a potential for greater clinical risk.
- The current evidence base remains limited, particularly in relation to human studies directly examining methamphetamine and its comparative harms.

**What are the implications of the main findings?**

- Recognising the pharmacological differences between methamphetamine and amphetamine may support clearer clinical and scientific assessment of methamphetamine-related harms.
- The limited human evidence highlights the need for further clinically relevant research to strengthen the evidence base in this area.

**Abstract:**

Methamphetamine presents a significant scientific and legal challenge for sentencing because, although it is a Class A drug in England and Wales, it is not assigned explicit indicative quantity thresholds within the principal Sentencing Council guideline. This review provides a comparative expert synthesis of methamphetamine in relation to amphetamine, cocaine and heroin, with particular emphasis on pharmacodynamics, pharmacokinetics, route-specific harms, fatal toxicity indicators and broader patterns of individual-harm profiles. The analysis draws on human laboratory studies, neuroimaging, pharmacokinetic investigations, toxicological literature, drug-related mortality data and policy sources to assess where methamphetamine most appropriately sits within a harm-based sentencing framework. The evidence indicates that methamphetamine is pharmacologically closest to amphetamine, sharing core monoaminergic mechanisms of transporter-mediated neurotransmitter release and vesicular disruption, but differing across several pharmacokinetic and toxicity-related parameters. Compared with amphetamine, methamphetamine shows greater lipid solubility, more efficient central nervous system penetration, longer persistence, and exposure that may, under common high-intensity routes of use, be associated with higher risk of neuropsychiatric, cardiovascular and cerebrovascular harms. Smoked methamphetamine in particular achieves high systemic bioavailability and rapid onset, creating a pattern of exposure more severe than conventional amphetamine preparations and, in some practical respects, closer to high-intensity stimulant models such as crack cocaine. Contemporary evidence further indicates that methamphetamine is associated with higher fatal toxicity than amphetamine, although the magnitude of difference varies by endpoint and no single universal gram-for-gram conversion is supported or recommended. Overall, the literature does not justify treating methamphetamine as simply equivalent to amphetamine, nor does it support conflating it fully with heroin or crack cocaine. The most defensible interpretation is that amphetamine should remain the primary scientific comparator, but with upward adjustment to reflect methamphetamine’s greater persistence and toxicity-related burden, while cocaine may serve as a secondary comparator for proportionality within the Class A sentencing framework. Taken together, the evidence supports consideration of an upward-adjusted amphetamine-based interpretation rather than an unadjusted amphetamine analogue.

## 1. Introduction and Background

Methamphetamine presents a distinctive challenge for sentencing guidance because it lies at an unusual intersection of pharmacology, toxicology and law. Under the United Kingdom (UK)’s Misuse of Drugs Act 1971, methamphetamine is classified as a Class A controlled drug, whereas amphetamine is generally treated as Class B and cocaine and heroin are Class A. For supply-type offences in England and Wales, however, sentencing is not determined by legal class alone: the Sentencing Council guidelines also use drug-specific quantity categories as proxies for harm. Unlike heroin, cocaine and amphetamine, methamphetamine is not assigned explicit indicative quantity thresholds within the principal guidelines. As a result, courts may rely more heavily on expert evidence to determine the most appropriate harm analogue. Any such analogue must be scientifically credible and legally proportionate [1,2,3].

Amphetamine-type stimulants (ATSs) are synthetic phenethylamine-derived central nervous system (CNS) stimulants whose principal effects arise from enhancement of monoaminergic neurotransmission, particularly dopamine (DA), norepinephrine (NE) and, to a lesser extent, serotonin (5-HT). Amphetamine and methamphetamine also act as monoamine releasers, with additional reuptake inhibition. This redistributes catecholamines from synaptic vesicles into the cytosol and facilitates reverse transport through plasma membrane monoamine transporters, thereby increasing extracellular monoamine concentrations. These actions underpin alerting and euphorigenic effects, but also sympathomimetic and neuropsychiatric toxicities including tachycardia, hypertension, agitation, hyperthermia, psychosis and seizures. Cocaine differs mechanistically because it acts primarily by blocking monoamine reuptake rather than by promoting vesicular redistribution and transporter-mediated monoamine efflux, although its additional sodium-channel effects are also relevant to cardiotoxicity [4,5,6,7,8,9].

Amphetamine is the most appropriate primary scientific comparator for methamphetamine because both compounds are structurally and pharmacodynamically closely related. Methamphetamine is the N-methyl analogue of amphetamine, and this apparently modest modification has important functional consequences. N-methylation increases lipid solubility, facilitates penetration across the blood–brain barrier (BBB), and enhances CNS delivery; together with differences in metabolic stability and exposure pattern, this is associated with more sustained central dopaminergic effects than are typically observed with amphetamine. In practical terms, methamphetamine is associated, under common high-intensity patterns of use, with greater risk of repeated use, dependence, psychosis and neurotoxicity relative to amphetamine. The Advisory Council on the Misuse of Drugs (ACMD)’s 2006 advice emphasised that, although methamphetamine is chemically related to amphetamine, its effects are more intense and, in policy terms, operationally distinct in important respects, particularly where smokable crystalline formulations are involved [2,10,11,12,13].

Route of administration is central to that distinction. Amphetamine in Europe and the UK is encountered predominantly as the sulphate salt, usually as a powder taken orally or intranasally, whereas methamphetamine may appear in powder, tablet or crystalline hydrochloride forms. Amphetamine sulphate is not commonly smoked because heating tends to degrade the substance, whereas methamphetamine hydrochloride, especially in crystalline form, can be efficiently vaporised and inhaled. The ACMD described the resulting effects as “crack-like”, although considerably longer lasting, and identified this smokability as an important aggravating feature of methamphetamine relative to amphetamine [2]. Human pharmacokinetic work supports the significance of this route: smoked methamphetamine achieves high systemic bioavailability and prolonged elimination, indicating a pattern of exposure that combines rapid delivery with sustained persistence [14]. In practical terms, this creates an exposure pattern more comparable to high-intensity stimulant use than to conventional amphetamine powder, even though methamphetamine remains pharmacologically closer to amphetamine than to cocaine [14,15].

Controlled human and neuroimaging data further reinforce the distinction between relatedness and equivalence. In a double-blind laboratory study, Kirkpatrick et al. (2012) found that intranasal methamphetamine and d-amphetamine produced broadly similar dose-related subjective effects, supporting the view that the two substances share important acute stimulant properties in humans [16]. However, this does not establish full equivalence across all endpoints relevant to sentencing in the UK. Subjective drug liking, physiological arousal, duration of action, route-specific reinforcement, neurotoxicity and long-term morbidity are not interchangeable domains. Cocaine remains a mechanistically distinct stimulant and not the closest scientific comparator, but it is still relevant as a secondary sentencing comparator because it is a listed Class A stimulant (Misuse of Drugs Act 1971) with defined quantity thresholds and because certain route-amplified harms of methamphetamine may be more coherently cross-checked against cocaine than against amphetamine alone [9,17,18].

Contemporary clinical and epidemiological evidence indicates that methamphetamine’s harms extend well beyond uncomplicated stimulant euphoria. Reviews show that cocaine and methamphetamine are both associated with arrhythmias, vasospasm, myocardial injury and sudden death, whereas methamphetamine is additionally associated with prolonged sympathomimetic stress, cerebrovascular injury, persistent neuropsychiatric sequelae and, in younger adults, stroke risk [19,20]. More recent European and clinical literature also emphasises that methamphetamine harms are strongly route- and context-dependent, including in chemsex, in which prolonged wakefulness, repeated dosing and disinhibition may magnify risk [13,21]. There is no simple unidimensional continuum against which these and other stimulants can be measured. Against this background, the scientific task is not to derive a false three-way equipotent conversion between amphetamine, methamphetamine and cocaine, but to determine where methamphetamine most plausibly sits, for sentencing purposes, relative to listed comparator drugs. In this review, human comparative pharmacology, route-specific pharmacokinetics, cardiovascular and neuropsychiatric harms, and modern harm-indicator literature are treated as the primary scientific basis for expert opinion, with older lethality data used only in a supportive context.

## 2. Aims and Objectives

The primary aim of this review was to develop a scientifically rigorous expert framework for evaluating methamphetamine in the context of UK sentencing guidance, where the substance is classified as a Class A drug but is not assigned explicit indicative quantity thresholds within the principal Sentencing Council drug supply guideline. The core question was, therefore, not merely whether methamphetamine is more harmful than amphetamine in a general pharmacological sense, but how the pattern of human harm associated with methamphetamine should be positioned relative to listed comparator drugs, particularly amphetamine as its closest structural analogue and cocaine as the most relevant listed Class A stimulant comparator for sentencing seriousness. In this context, the review sought to determine whether the available scientific evidence supports direct equivalence with amphetamine, upward adjustment beyond amphetamine, or a secondary seriousness cross-check against cocaine-related quantity categories, while explicitly avoiding unsupported gram-for-gram interchangeability claims [2,3,12,13,22].

More specifically, the review aimed to compare amphetamine and methamphetamine across the domains most relevant to sentencing-related harm assessment: chemical and physicochemical properties; pharmacodynamics at monoamine transporters and vesicular systems; route-specific pharmacokinetics, especially intranasal and smoked administration; duration of CNS exposure; acute cardiovascular and neuropsychiatric toxicity; dependence-related and neurotoxic potential; and available lethality or fatal-toxicity indicators. A secondary objective was to examine the extent to which human comparative evidence, including controlled laboratory studies, neuroimaging findings and contemporary harm literature, can support a principled sentencing analogue for methamphetamine in the absence of a dedicated guideline threshold. The review also aimed to identify where the literature remains insufficient for precise equipotency calculations and where expert judgment must, therefore, remain qualified and endpoint-specific [16,18,19,20,23].

Accordingly, the operational objectives of the review were fourfold: first, to synthesise the comparative scientific literature on amphetamine, methamphetamine and, where relevant, cocaine, with particular emphasis on human evidence; second, to organise that literature into a sentencing-relevant evidence hierarchy that distinguishes mechanistic similarity from broader harm seriousness; third, to evaluate proposed comparative frameworks, including conservative and upward-adjusted scenarios, without overstating the certainty of any single ratio or conversion; and fourth, to provide a transparent expert basis for discussing where methamphetamine may most appropriately sit, in harm terms, relative to existing UK sentencing guideline categories for amphetamine and cocaine, while recognising heroin as an upper-end reference point for overall seriousness under UK law [2,3,24].

## 3. Methods

### 3.1. Study Design

This study was conducted as a narrative expert synthesis and comparative literature review based exclusively on secondary sources. No primary data collection, experimental work, laboratory testing, forensic re-analysis or original toxicological modelling was undertaken. The approach was selected because the underlying question is interpretive and translational rather than purely empirical: the objective was to evaluate how existing pharmacological, toxicological and harm-related evidence can inform sentencing treatment of an unlisted drug, rather than to generate a pooled quantitative estimate of biological effect. A narrative expert method was therefore considered appropriate, although such an approach is inherently more susceptible to selection bias and less reproducible than a formal systematic review. Formal meta-analysis was limited, and in some domains precluded, by marked heterogeneity in study designs, endpoints, routes of administration and evidential domains relevant to the question [25,26].

### 3.2. Literature Identification

Literature identification was undertaken through targeted review of peer-reviewed and authoritative grey-literature sources relevant to stimulant pharmacology, comparative toxicity, human laboratory drug effects, route-specific pharmacokinetics, neuroimaging, fatal-toxicity indicators and UK drug-policy context. Searches were conducted in PubMed, Scopus and Google Scholar up to March 2026, supplemented by targeted searching of official policy and legal sources, including ACMD reports, Sentencing Council materials, UK legislation and European Union Drugs Agency (EUDA) resources. Search combinations included terms such as “methamphetamine AND pharmacokinetics”, “methamphetamine AND fatal toxicity”, “amphetamine AND methamphetamine comparison”, “methamphetamine AND neuroimaging”, and “methamphetamine AND sentencing”. Core source domains included historical and contemporary studies, and official UK policy materials, including ACMD reports and Sentencing Council guidance [2,3,7,12,13,16,18,19,20,26]. Because this was a narrative expert review rather than a prospectively registered systematic review, a formal PRISMA flow diagram and full screening log were not maintained; this should be regarded as a methodological limitation.

### 3.3. Eligibility Criteria

Sources were considered eligible if they addressed one or more of the following domains: pharmacodynamics, including monoamine release, transporter reversal, vesicular monoamine storage disruption or reuptake inhibition; pharmacokinetics, including half-life, route-specific absorption, bioavailability, brain uptake or persistence; comparative human data, such as laboratory self-administration, subjective effects, physiological effects or neuroimaging; toxicity and harm metrics, including acute lethality, fatal-toxicity indicators, poisoning deaths, cardiovascular or cerebrovascular complications, and multidimensional harm rankings; or legal and policy context relevant to UK drug classification and sentencing. Sources were excluded where they were clearly not relevant to comparative stimulant harms, were linguistically non-English, or offered only isolated anecdotal material without broader interpretive value. Animal-only behavioural studies with limited translational relevance to human harm assessment were deprioritised rather than categorically excluded. Case reports and post-mortem data were used cautiously and only where they contributed to a wider evidential pattern rather than as standalone determinants of comparative sentencing seriousness.

### 3.4. Data Extraction

Data extraction focused on both quantitative and qualitative elements relevant to the review question. Quantitative elements included half-life estimates, route-specific bioavailability, duration of subjective and physiological effects, human study dose ranges, fatal toxicity indicators and drug-related mortality measures, multidimensional harm scores and indicative sentencing quantities for listed drugs under the Sentencing Council framework. Qualitative elements included route-specific patterns of use, mechanism-based distinctions between monoamine releasers and reuptake inhibitors, evidence of longer CNS persistence, comparative neurotoxicity, and policy statements regarding methamphetamine’s classification and harms. Extraction was not undertaken as a formal tabulated risk-of-bias exercise, because the included materials spanned different evidence types, from controlled human laboratory studies to policy reports and toxicological syntheses. Instead, emphasis was placed on evidential relevance, methodological credibility, and direct applicability to the sentencing question.

### 3.5. Synthesis Strategy

The synthesis was undertaken thematically. The first stage examined chemical and mechanistic distinctions between amphetamine, methamphetamine and cocaine, so as to identify the most defensible primary scientific comparator. The second stage evaluated pharmacokinetic and route-related evidence, especially where smoked and intranasal administration altered onset, intensity, persistence or abuse liability. The third stage considered human toxicity, morbidity and fatal-toxicity indicators, alongside multidimensional harm frameworks, to assess whether methamphetamine’s overall seriousness diverges materially from that of amphetamine. The fourth stage translated these findings into sentencing relevance by considering methamphetamine against the existing indicative quantity thresholds for amphetamine, cocaine and heroin, while explicitly recognising that no precise equipotent conversion is validated in the literature. Proposed comparative scenarios, including conservative and upward-adjusted interpretations, were therefore treated as structured expert frameworks rather than exact biological conversion formulae. Specifically, the comparative scenarios discussed in this review comprised a conservative 1:1 scenario, a moderate fatal-toxicity adjustment scenario (approximately 1.4-fold), a stronger toxicity-based scenario (approximately two-fold), and a route-sensitive pharmacokinetic amplification scenario. These were derived from the literature using stepwise consideration of acute lethality data, fatal-toxicity differentials, route-specific bioavailability and review-based potency estimates, with explicit recognition that they do not represent fixed equipotent or interchangeable gram-for-gram conversions [3,14,16,22,24,27,28].

## 4. Results

### 4.1. Comparative Pharmacology and Harm Profile

Methamphetamine and amphetamine are closely related ATS, but the reviewed evidence indicates that methamphetamine is associated with higher risk across several clinically and toxicologically relevant domains. Both drugs act primarily as indirect sympathomimetic monoamine releasers, increasing extracellular dopamine, noradrenaline and serotonin through actions at DAT, NET and SERT and through disruption of vesicular monoamine storage via VMAT2 [4,5,7]. By redistributing vesicular monoamines into the cytosol, VMAT2 disruption facilitates reverse transport through plasma membrane monoamine transporters and thereby amplifies extracellular catecholamine exposure. Methamphetamine’s additional N-methyl group increases lipid solubility and facilitates more efficient penetration across the BBB, a property likely to contribute to greater intensity and duration of central dopaminergic exposure, although these effects are also influenced by metabolism, dose and route of administration [7,10]. Consistent with these pharmacological differences, the clinical literature more often associates methamphetamine, particularly under chronic or high-intensity patterns of use, with persistent neuropsychiatric sequelae, severe cardiovascular complications, cerebrovascular injury and compulsive patterns of repeated use [10,19,20,29].

### 4.2. Human Comparative Evidence

The controlled human evidence does not support the proposition that methamphetamine and d-amphetamine are qualitatively different drugs in every acute experimental respect, but it equally does not support simple equivalence in harm terms. In the key double-blind intranasal comparison by Kirkpatrick et al. (2012), methamphetamine and d-amphetamine produced a similar dose-related profile of self-administration, subjective effects and physiological responses, supporting their close pharmacological relatedness [16]. Nevertheless, that study did not establish identity across endpoints relevant to sentencing, including duration of action, cumulative toxicity, route-amplified reinforcement and longer-term morbidity. The human imaging literature further distinguishes the two drugs from cocaine in important ways: Fowler et al. (2008) demonstrated that methamphetamine enters the human brain rapidly but remains in brain tissue substantially longer than cocaine, with peak uptake at approximately 9 min and prolonged retention in cortical and subcortical regions [18]. Taken together, these findings support a nuanced interpretation: methamphetamine is clearly amphetamine-like in core stimulant pharmacology, but its duration, persistence and broader morbidity profile make it more harmful than a simple acute subjective-effect comparison might imply [16,18].

### 4.3. Route-Specific Pharmacokinetics

Route of administration is a critical determinant of comparative harm. Methamphetamine hydrochloride can be efficiently smoked, and this has long been recognised by UK policy bodies as an important distinction from amphetamine. The ACMD report stated in 2006 that methylamphetamine hydrochloride is smokable and, when used in that fashion, can produce effects similar to crack cocaine, though considerably longer lasting [2]. Human pharmacokinetic data support the significance of this route. In the study by Cook et al. (1993), the bioavailability of smoked methamphetamine was 90.3 ± 10.4%, whereas oral bioavailability was 67.2 ± 3.1%; the geometric mean plasma half-life was 11.1 h following smoking and 12.2 h following intravenous administration [14]. These findings indicate that smoking permits both high systemic exposure and prolonged elimination. By contrast, cocaine typically has a much shorter half-life, often around 40 to 90 min, contributing to rapid cycling and repeated dosing rather than prolonged persistence [9]. Although amphetamine may also be used orally or intranasally with substantial bioavailability, the reviewed literature does not support the same degree of efficient smoking-based delivery that characterises crystalline methamphetamine [2,9,10,14].

### 4.4. Acute Lethality and Fatal Toxicity

Acute toxicity metrics also place methamphetamine above amphetamine, although the magnitude of difference depends on the endpoint selected. Historical animal lethality data cited in the toxicological literature indicate substantially lower LD_50_ values for methamphetamine than for amphetamine. In dogs, oral LD_50_ values of approximately 11.5 mg/kg for methamphetamine and 23.3 mg/kg for amphetamine, and intravenous values of approximately 2.95 mg/kg and 5.9 mg/kg, respectively, have often been interpreted as indicating an approximately two-fold difference in acute lethality under experimental conditions [28]. These animal-derived data are directionally supportive only and are not determinative of human comparative toxicity. More recent comparative fatal-toxicity work provides a population-based signal in the same direction. Kriikku et al. (2024), using poisoning deaths in conjunction with wastewater-based consumption estimates, reported a higher fatal-toxicity indicator for methamphetamine than for amphetamine at an assumed 50 mg dose, while both remained within broadly similar stimulant range rather than representing wholly separate toxicological classes [24]. This contemporary evidence supports an upward adjustment from amphetamine rather than strict equivalence, while also indicating that methamphetamine belongs within a stimulant harm spectrum that is more plausibly cross-checked against cocaine than against a lower-harm Class B (Misuse of Drugs Act 1971) baseline alone [24,27].

### 4.5. Cardiovascular and Cerebrovascular Morbidity

The modern clinical literature strengthens the case for distinguishing methamphetamine from amphetamine by showing that harm is not limited to acute overdose lethality. Methamphetamine and cocaine are both associated with major cardiovascular toxicity, including tachyarrhythmias, hypertension, vasospasm, myocardial injury and sudden death, but methamphetamine’s longer duration introduces a prolonged period of sympathomimetic stress that may amplify cumulative harm [19]. Recent reviews also identify methamphetamine as an important cause of stroke in younger populations, with both haemorrhagic and ischaemic mechanisms implicated, including severe hypertension, vasculopathy, vasospasm, cardiac dysrhythmia and accelerated vascular injury [20]. These end-organ harms are not adequately captured by acute lethality metrics alone and are particularly relevant to sentencing because they extend beyond transient intoxication to serious downstream morbidity, including stroke, cardiomyopathy and persistent psychiatric injury. Although amphetamine can also cause severe acute toxicity, the reviewed evidence indicates that methamphetamine is more consistently associated with severe and prolonged downstream injury [10,19,20].

### 4.6. Multi-Criteria Harm Rankings

Broader harm-ranking studies place methamphetamine among the most harmful drugs to the individual. In the UK multicriteria decision analysis by Nutt et al. (2010), heroin, crack cocaine and methamphetamine were the three most harmful drugs to individuals, with part scores of 34, 37 and 32, respectively [22]. Amphetamine ranked lower on this individual-harm dimension, and cocaine also ranked below crack and methamphetamine in overall individual severity. These data do not function as sentencing tables and should not be treated as such. They are, however, highly informative because they show that when multiple domains of harm are considered together, methamphetamine is positioned substantially above amphetamine and nearer the upper tier of seriously harmful stimulants. Although these harm-ranking studies are partly based on expert consensus and subjective weighting methodologies, these findings are consistent with the conclusions from the pharmacological and toxicological literature that methamphetamine should not be approached as merely a more concentrated form of amphetamine [22].

### 4.7. Implications for Comparison with Current UK Sentencing Thresholds

Within the current Sentencing Council framework, methamphetamine presents a clear anomaly: it is a Class A drug but does not have explicit indicative quantity thresholds in the principal UK sentencing guideline, whereas heroin and cocaine have thresholds of 150 g for Category 3, 1 kg for Category 2 and 5 kg for Category 1, and amphetamine has thresholds of 750 g for Category 3, 4 kg for Category 2 and 20 kg for Category 1 [3]. The scientific findings above indicate that amphetamine is the correct primary comparator because it is the closest structural and mechanistic analogue, but they also indicate that methamphetamine’s greater potency, greater route-amplified exposure, longer persistence and greater harm burden justify a more serious assessment than an unadjusted amphetamine comparison alone. Cocaine remains relevant as a secondary comparator because it is a listed Class A stimulant and because some harms of smoked methamphetamine, especially rapid onset and severe cardiovascular strain, are more coherently assessed against cocaine than against conventional amphetamine powder [2,3].

### 4.8. Comparative Scenario Framework

The reviewed literature does not support a formal bioequivalence model between methamphetamine and amphetamine in the pharmaceutical or regulatory sense, and any attempt to express the relationship as a single fixed gram-for-gram conversion would create false precision. Rather, the available evidence supports a set of comparative analytical scenarios that reflect different evidential endpoints relevant to sentencing seriousness. In this context, the scenarios considered in this review should be understood as structured harm-comparison frameworks, each anchored to a distinct scientific domain: controlled human acute effects, fatal toxicity, experimental acute lethality, and route-specific pharmacokinetic amplification.

The most conservative scenario is the 1:1 framework, derived principally from the controlled human findings of Kirkpatrick et al. (2012), in which intranasal methamphetamine and d-amphetamine produced broadly similar dose-related subjective, reinforcing and physiological effects [16]. This scenario confirms that methamphetamine remains pharmacodynamically close to amphetamine and cannot be treated as a wholly separate stimulant class. However, it provided a lower-bound comparator rather than a complete account of relative harm seriousness because it does not incorporate smoked exposure, prolonged brain persistence, cumulative neurotoxicity, downstream vascular injury or broader fatal-toxicity indicators [14,16].

A second, moderate-adjustment scenario is represented by the 1:1.4 framework, reflecting comparative fatal-toxicity indicators rather than dose equivalence. This should be understood as a fatal-toxicity-related differential rather than a dose-equivalence ratio. Moreover, this is based principally on Kriikku et al. (2024), in which methamphetamine showed a higher fatal-toxicity indicator than amphetamine under the modelled 50 mg assumption [24]. A third scenario, approximately 2:1, is based on historical acute lethality evidence indicating substantially lower LD_50_ values for methamphetamine than for amphetamine in experimental models. These data support the direction of effect, namely that methamphetamine is more acutely toxic than amphetamine, although they are not sufficient to justify direct translation into human sentencing equivalence. A fourth, route-sensitive pharmacokinetic amplification scenario reflects the fact that smoked methamphetamine can achieve very high systemic bioavailability and prolonged persistence, thereby amplifying seriousness under high-intensity exposure conditions. This should not be interpreted as a universal potency ratio or a fixed conversion model.

Taken together, these scenarios do not identify a single definitive equivalence factor. Instead, they demonstrate that the answer depends on which scientific endpoint is given primacy. Acute controlled human effects support close relatedness and a conservative lower-bound interpretation, fatal-toxicity indicators support moderate upward adjustment, historical lethality data support a stronger toxicological distinction, and route-specific pharmacokinetic evidence supports the view that, under some exposure conditions, methamphetamine may behave substantially more seriously than ordinary amphetamine. The collective implication is that methamphetamine cannot be defended scientifically as simply equivalent to amphetamine across all relevant domains [14,16,24,27,28].

## 5. Discussion and Expert Opinion

### 5.1. Scientific Basis for Distinguishing Methamphetamine from Amphetamine

The central scientific issue is not whether methamphetamine belongs to the amphetamine family, but whether that relationship is sufficient to justify treating it as ordinary amphetamine for sentencing purposes. The literature indicates that it is not. Methamphetamine is the N-methyl analogue of amphetamine and shares its core monoaminergic mechanism, but that structural modification has important functional consequences, including greater lipid solubility, more efficient penetration of the BBB, and more sustained CNS exposure [4,10]. As with amphetamine, its pharmacodynamic effects depend on transporter-mediated uptake into presynaptic neurones, disruption of vesicular monoamine storage through VMAT2, redistribution of dopamine and noradrenaline into the cytosol, and reverse transport through plasma membrane monoamine transporters. However, methamphetamine appears to generate a more intense and prolonged dopaminergic burden and is associated, particularly under common high-intensity patterns of use, with greater risk of repeated use, dependence, psychosis and neurotoxicity relative to amphetamine [5,7]. The ACMD’s 2006 advice described methamphetamine’s effects as more intense than amphetamine’s and, in policy terms, different in important operational respects, with smokability identified as a particularly important aggravating feature because it could produce crack-like effects of longer duration [2]. Taken together, the pharmacological and policy literature supports distinguishing methamphetamine from amphetamine in sentencing terms, even while recognising their close scientific relationship.

### 5.2. Mechanism Versus Harm

Mechanistic similarity alone is insufficient for sentencing analysis. Amphetamine and methamphetamine are both monoamine-releasing stimulants, whereas cocaine acts primarily through monoamine reuptake inhibition [7,9]. On a purely mechanistic basis, amphetamine is, therefore, the closest comparator. However, sentencing is concerned with harm caused rather than receptor classification alone. Once the analysis is expanded to include persistence, route-specific exposure, fatal toxicity and severe downstream morbidity, the case for simple amphetamine equivalence weakens considerably. Methamphetamine is associated with higher smoked bioavailability, prolonged central exposure, stronger evidence of neurotoxicity, and greater fatal toxicity than amphetamine in recent wastewater-linked mortality analyses [14,19,24]. The scientific difficulty for sentencing, therefore, lies precisely in this divergence: methamphetamine remains amphetamine-like in mechanism, but not amphetamine-equivalent in seriousness.

For sentencing purposes, the comparative scenarios considered in this review should be understood as analytical frameworks rather than literal conversion formulae. The 1:1, 1:1.4, 2:1 and 1:2.46 scenarios arise from different evidential anchors, including shared pharmacodynamic effects, fatal-toxicity differentials, historical acute lethality data, and route-specific pharmacokinetic amplification. Their purpose is not to assert a single universal gram-for-gram equivalence between methamphetamine and amphetamine, but to test where methamphetamine most plausibly sits on a harm continuum when multiple domains of evidence are considered together [14,16,24,27,28].

#### 5.2.1. Critical Appraisal of a Strict 1:1 Approach

A strict 1:1 analogy with amphetamine is scientifically defensible only as a deliberately conservative lower-bound position. Its principal support lies in the controlled human study by Kirkpatrick et al. (2012), which showed that intranasal methamphetamine and d-amphetamine generated broadly similar dose-related subjective, reinforcing and physiological effects [16]. This is important evidence because it demonstrates that methamphetamine does not occupy a wholly separate pharmacodynamic category from amphetamine and that direct human comparison does not support exaggerated claims of absolute divergence. In expert terms, the study is valuable because it anchors the analysis in human data rather than relying exclusively on toxicological inference or policy analogy [16].

Nevertheless, the evidential weight of a strict 1:1 approach is limited by the narrowness of the endpoints from which it is derived. Acute subjective and physiological similarity under controlled intranasal administration does not establish equivalence across the broader domains that matter to sentencing seriousness. It does not capture smoked exposure, prolonged systemic persistence, longer brain residence, cumulative binge-pattern use, severe downstream vascular sequelae, or comparative fatal toxicity [14,18,24]. Nor does it adequately reflect the long-recognised policy and clinical distinction that methamphetamine, especially in smokable crystalline form, presents a materially aggravated harm profile compared with ordinary amphetamine preparations [2,10].

For those reasons, the 1:1 framework should not be discarded, but nor should it be elevated into a complete scientific account of relative seriousness. Its proper role is as a reference point demonstrating pharmacological relatedness and setting the floor below which interpretation should not fall. In other words, it can justify the proposition that amphetamine is the correct comparator family, but not the stronger proposition that methamphetamine should be treated as fully equivalent to amphetamine in sentencing seriousness.

#### 5.2.2. Critical Appraisal of a Moderate Upward Adjustment

A moderate upward adjustment from amphetamine is, in our view, the most balanced interpretation of the current evidence base. This conclusion does not depend on any claim of exact quantitative conversion. Rather, it reflects the convergence of several different lines of evidence, each of which points in the same direction. Human pharmacokinetic work demonstrates that smoked methamphetamine can achieve high systemic bioavailability together with prolonged elimination [14]. Neuroimaging work shows rapid brain uptake and persistence substantially longer than cocaine [18]. Comparative fatal-toxicity work indicates that methamphetamine is more toxic than amphetamine in real-world mortality terms [24]. The clinical literature further associates methamphetamine with severe and persistent neuropsychiatric, cardiovascular and cerebrovascular harms that exceed what would ordinarily be inferred from a simple amphetamine-equivalence model [19,20,29].

What makes this middle position especially persuasive is that it avoids the main weaknesses at either end of the interpretive spectrum. It avoids the underinclusive nature of a strict 1:1 model, which is too dependent on limited acute laboratory endpoints, but it also avoids overreliance on animal lethality or highly contingent route-based multipliers as the sole basis for judgment. A moderate upward adjustment therefore best reflects the totality of the evidence: methamphetamine is not merely a nominally stronger version of amphetamine, but neither does the literature justify presenting it as universally convertible by a single large multiplier across all contexts [14,18,19,20,24].

In expert-opinion terms, this is the most defensible interpretive position because it is both evidence-responsive and methodologically restrained. It acknowledges the absence of true bioequivalence data, resists false mathematical precision, and yet still gives due weight to the fact that the broader harm profile of methamphetamine is materially more serious than that of unadjusted amphetamine.

#### 5.2.3. Critical Appraisal of Stronger Toxicity-Based Scenarios

Stronger toxicity-based scenarios remain scientifically relevant, but they should be treated with greater caution. Historical lethality studies indicate that methamphetamine may be substantially more acutely toxic than amphetamine under experimental conditions, and route-sensitive pharmacokinetic reasoning suggests that smoking can generate a high-intensity exposure profile that meaningfully amplifies seriousness. These findings are not trivial and should not be dismissed. They are important because they test the upper range of plausible distinction and demonstrate that a simple amphetamine-equivalence model may substantially underestimate seriousness under some conditions [14,28].

At the same time, these scenarios are methodologically less secure as primary sentencing anchors. Animal LD_50_ data do not translate directly into human equivalence because they abstract away from route variability, tolerance, polydrug exposure, individual vulnerability, purity and emergency treatment. Similarly, route-amplified pharmacokinetic frameworks may overstate general comparability if applied indiscriminately to cases in which the intended route of administration is uncertain or where the seized material cannot reliably be linked to smoking-based use [14,28]. Such scenarios therefore have greater value as sensitivity analyses or upper-bound interpretive models than as universal comparative rules.

Their proper scientific role is to demonstrate the limits of conservative interpretation. They show that the seriousness of methamphetamine can, under certain toxicological or route-specific assumptions, diverge markedly from that of amphetamine. However, because these frameworks are more contingent and less directly generalisable than the broader convergent evidence supporting moderate upward adjustment, they should inform but not dominate the expert conclusion. In our view, they strengthen the argument against strict equivalence, but they do not justify replacing a qualified, evidence-balanced interpretation with a single maximal multiplier.

### 5.3. The Role of Cocaine as a Secondary Comparator

Cocaine should not replace amphetamine as the primary mechanistic comparator, but it remains highly relevant as a secondary comparator for sentencing seriousness. Mechanistically, cocaine is distinct, acting predominantly as a reuptake inhibitor and having a much shorter half-life than methamphetamine [9]. However, smoked methamphetamine shares with crack cocaine certain practical features of high-harm stimulant exposure: rapid delivery, intense reinforcement, acute cardiovascular stress and high-risk patterns of repeated dosing. The ACMD explicitly highlighted this in 2006 when it described smokable methamphetamine as capable of producing crack-like effects, though considerably longer lasting. In sentencing terms, this matters because the guideline already contains quantity categories for cocaine but not for methamphetamine. Cocaine, therefore, provides a useful Class A seriousness cross-check, especially where the concern is not mechanistic equivalence but proportional placement within an existing quantity-based framework [2,3].

### 5.4. Position Relative to Heroin and Crack Cocaine

The evidence does not support treating methamphetamine as interchangeable with heroin or crack cocaine, but it equally does not support minimising its seriousness by aligning it too closely with amphetamine alone. Heroin remains toxicologically distinct as an opioid agonist and appears in comparative lethality reviews as one of the most directly physiologically toxic drugs, particularly by intravenous use [27]. In Nutt et al.’s (2010) multicriteria decision analysis, heroin and crack cocaine remained the most harmful substances overall, and both exceeded methamphetamine when harms to others and total aggregate harms were considered, even though methamphetamine ranked very highly for harm to the individual [22]. The most scientifically accurate position is, therefore, an intermediate but serious one: methamphetamine lies above amphetamine and below heroin and crack cocaine in overall harm severity, while approaching cocaine and, in some individual-harm dimensions, exceeding it. That is precisely the pattern that makes a moderate-to-strong upward adjustment from amphetamine scientifically defensible and a cocaine-based Class A cross-check policy-coherent.

### 5.5. Expert Opinion

On the basis of the reviewed evidence, the most balanced interpretation is that methamphetamine should be benchmarked primarily against amphetamine because of its close structural and pharmacodynamic relationship, but that benchmark should be adjusted upward to reflect materially greater harm. The evidence supporting adjustment includes higher smoked bioavailability, longer elimination half-life in smoking studies, greater CNS penetration, longer brain persistence, higher fatal toxicity than amphetamine in modern wastewater-linked mortality analyses, and stronger evidence of severe neurotoxic, cardiovascular and cerebrovascular morbidity [14,18,19,20,24]. Cocaine should be used as a secondary comparator for proportionality within the Class A sentencing framework, especially where route-related harms suggest a high-intensity stimulant exposure model. The literature does not support a single universal gram-for-gram conversion, and any attempt to present one as exact would create false precision. It does, however, support a clear qualitative conclusion: methamphetamine is materially more harmful than amphetamine and should not be assessed on an unadjusted amphetamine-equivalent basis for sentencing purposes. On balance, the reviewed evidence supports an upward-adjusted amphetamine-based interpretation, with cocaine Category 2 serving as a pragmatic secondary proportionality cross-check within the current Class A framework.

## 6. Conclusions and Limitations

Methamphetamine is pharmacologically closest to amphetamine, but the available evidence indicates meaningful difference in pharmacokinetics, persistence, and several toxicity-related parameters. Compared with amphetamine, methamphetamine is associated, particularly under common high-intensity patterns of use, with higher risk of neuropsychiatric, cardiovascular and cerebrovascular harm. These differences are especially relevant where smoked or intranasal administration is contemplated, because rapid delivery can increase reinforcing effects and acute toxicity.

Taken together, the literature supports a proportionate upward adjustment from an amphetamine-based benchmark rather than a strict 1:1 equivalence. At the same time, the evidence does not justify treating methamphetamine as fully interchangeable with heroin or crack cocaine. Amphetamine remains the primary scientific comparator, while cocaine serves as a useful secondary Class A comparator for proportionality, particularly in relation to route-amplified stimulant harms and the absence of explicit methamphetamine quantity thresholds in current sentencing guidance.

Accordingly, methamphetamine should be regarded as a high-harm stimulant that sits materially above amphetamine in seriousness, but below heroin and crack cocaine in overall harm. No single gram-for-gram conversion is supported by the evidence, and any ratio should be presented as an expert analytical aid rather than as an exact biological equivalence. In policy terms, the evidence does not support treatment of methamphetamine as an unadjusted amphetamine analogue.

This review is based on secondary literature and does not include new primary laboratory, clinical, neuroimaging or forensic data. Human head-to-head comparative studies remain limited, route-specific pharmacokinetic data for smoked methamphetamine are relatively sparse, and some toxicity arguments still rely partly on animal-derived lethality data that cannot be translated directly into human sentencing equivalence. In addition, real-world harms vary according to context and setting, idiosyncratic reactions, purity, route of use, tolerance, co-morbidity, co-ingested substances and emergency response, and these factors cannot always be inferred from seizure data alone. The conclusions should, therefore, be read as a structured expert synthesis supporting proportionate sentencing judgment, rather than as a fixed mathematical conversion model.

## Data Availability

No new data were created or analysed in this study. Data sharing is not applicable to this article.
